# Sarcopenia in Chronic Kidney Disease: Focus on Advanced Glycation End Products as Mediators and Markers of Oxidative Stress

**DOI:** 10.3390/biomedicines9040405

**Published:** 2021-04-09

**Authors:** Elena Dozio, Simone Vettoretti, Giuseppe Lungarella, Piergiorgio Messa, Massimiliano M. Corsi Romanelli

**Affiliations:** 1 Department of Biomedical Science for Health, Laboratory of Clinical Pathology, Università degli Studi di Milano, 20133 Milan, Italy; mmcorsi@unimi.it; 2 Unit of Nephrology, Dialysis and Kidney Transplantation, Fondazione IRCCS Ca’ Granda Ospedale Maggiore Policlinico di Milano, 20122 Milan, Italy; simone.vettoretti@policlinico.mi.it (S.V.); piergiorgio.messa@unimi.it (P.M.); 3 Department of Molecular and Developmental Medicine, Università di Siena, 53100 Siena, Italy; lungarella.giuseppe@gmail.com; 4 Department of Clinical Science and Community Health, Università degli Studi di Milano, 20133 Milan, Italy; 5 Service of Laboratory Medicine1-Clinical Pathology, IRCCS Policlinico San Donato, San Donato Milanese, 20097 Milan, Italy

**Keywords:** chronic kidney disease, advanced glycation end products (AGE), oxidative stress, sarcopenia

## Abstract

Sarcopenia is common in chronic kidney disease (CKD), and it is independently associated with morbidity and mortality. Advanced glycation end products (AGE) are mainly known as aging products. In CKD, AGE accumulate due to increased production and reduced kidney excretion. The imbalance between oxidant/antioxidant capacities in CKD patients is one of the main factors leading to AGE synthesis. AGE can, in turn, promote CKD progression and CKD-related complications by increasing reactive oxygen species generation, inducing inflammation, and promoting fibrosis. All these derangements can further increase AGE and uremic toxin accumulation and promote loss of muscle mass and function. Since the link between AGE and sarcopenia in CKD is far from being fully understood, we revised hereby the data supporting the potential contribution of AGE as mediators of oxidative stress in the pathogenesis of sarcopenia. Understanding how AGE and oxidative stress impact the onset of sarcopenia in CKD may help to identify new potential markers of disease progression and/or therapeutic targets.

## 1. Sarcopenia in Chronic Kidney Disease: Potential Mechanisms

Sarcopenia is a condition characterized by progressive loss of skeletal muscle mass, strength, and function. Although sarcopenia is primarily associated with aging, it can be observed in different pathological conditions independent of age, among which chronic kidney disease (CKD) plays a prominent role. In advanced CKD, this association led to the definition of “uremic sarcopenia” [1]. However, sarcopenia occurs at all stages of CKD, its severity increases with the decreasing of renal function [2], and it correlates with physical disability, poor quality of life, increased cardiovascular risk, and overall mortality [3,4,5]. Consequently, early diagnosis and effective therapeutic interventions to avoid sarcopenia-related complications have become a clinical priority.

In patients affected by CKD, the onset of sarcopenia is a complex phenomenon. Its etiology has been associated with numerous mechanisms, such as changes of hormonal signals (i.e., insulin/insulin-like growth factors, growth hormone, vitamin D, sex hormones, myostatin, and angiotensin II); increased levels of inflammatory cytokines (i.e., tumor necrosis factor-α, interleukin-6 and -1α); reduced protein intake, myocellular changes (ATP and glycogen depletion, alteration in oxygen transport due to anemia); reduced function of satellite cells; metabolic acidosis; electrolyte disorders; alteration of hypothalamic orexigenic neuropeptides; and physical inactivity. Overall, the net effects of these mechanisms are decreased protein synthesis, increased muscle proteolysis, and reduced muscle strength [6,7,8]. The maintenance of muscle mass is a balance between protein synthesis and degradation. Therefore, any condition increasing protein degradation or decreasing protein synthesis may affect muscle mass. From a molecular point of view, protein degradation may be induced via the ubiquitin–proteasome system (UPS) and autophagy. In CKD, increased oxidative stress, inflammation, protein-bound uremic toxins, defective insulin signaling, parathyroid hormone, glucocorticoid, and angiotensin II are some of the key mediators that can initiate the above-mentioned pathways. Interestingly, myostatin, an autocrine inhibitor of muscle growth that is mainly produced in skeletal muscle, can induce muscle loss, too, and its levels have been shown to be upregulated by oxidative stress in CKD [9].

Previous studies conducted in settings different from CKD [10,11,12,13] demonstrated that the accumulation of advanced glycation end products (AGE) was strongly related to oxidative stress and could impact muscle function. Thus, it is plausible that AGE may contribute to the onset and progression of sarcopenia in CKD as well [14]. However, until now, this association has been poorly explored.

In this review, we aimed to summarize the studies that investigated the association between AGE as mediators of oxidative stress and the development of sarcopenia in patients affected by CKD. Understanding how AGE and oxidative stress impact the onset of sarcopenia in CKD may help to identify new potential markers of disease progression and/or therapeutic targets.

## 2. AGE in CKD: Pathogenetic Role

### 2.1. AGE Synthesis and Accumulation

**Figure 1:**
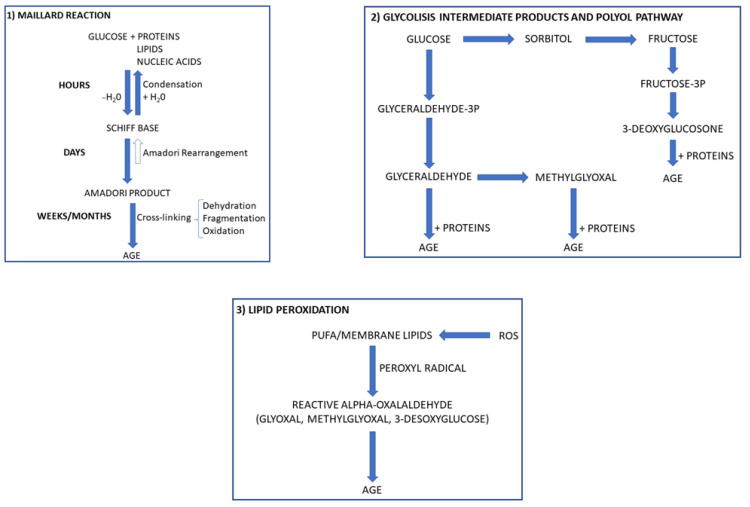
Biochemical reactions leading to advanced glycation end product (AGE) formation. Glucose may lead to AGE formation through the Maillard reaction (**1**) and the polyol pathway (**2**). This last mainly occurs in conditions of excessive glucose, which is converted into fructose. AGE can also be formed by reactive carbonyl species generated by lipid peroxidation in conditions of excessive oxidative stress and further reactions with nucleophilic residues of macromolecules (**3**). ROS, reactive oxygen species; PUFA, polyunsaturated fatty acids.

AGE are a heterogeneous group of irreversible products which comprise fluorescent cross-linking AGE (i.e., vesperlysine, pentosidine, and crossline), non-fluorescent cross-linking AGE (i.e., imadazolium dilysine cross-links, alkyl formyl glycosyl pyrroles, and arginine-lysine imid-azole cross-links), and non-cross-linking AGE (i.e., N-fructosyl-lysine, N carboxyethyl-lysine, and N-carboxymethyllysine). The formation of such products may occur through the non-enzymatic glycation and the glycoxidation of proteins, lipids, and nucleic acids [15,16] (Figure 1).

Non-enzymatic glycation is a complex process in which proteins, lipids, and nucleic acids react with reducing sugars and their metabolites. Structural proteins such as collagen and elastin, apoB-lipoprotein, fibrinogen, and albumin are potential targets of the glycation process. Synthesis of AGE through glycoxidation involves reactive oxygen species (ROS), which promote the synthesis of highly reactive carbonyl intermediates such as glyoxal and methyl-glyoxal. These compounds may further react with different biomolecules to produce AGE [15] (Figure 1). With aging, we can observe a physiological accumulation of these products, which are highly detrimental because they can promote inflammation and work as stressors for many organs. In specific pathological conditions characterized by an increased availability of substrates such as glucose and ROS, AGE formation is accelerated [16,17].

**Figure 2:**
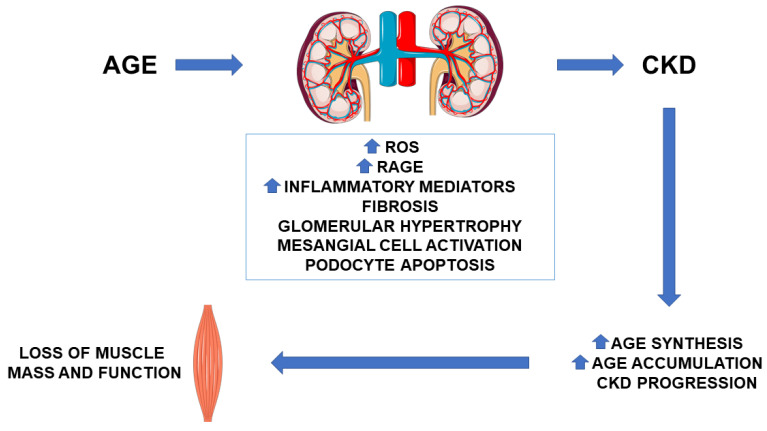
Advanced glycation end products (AGE) in chronic kidney disease (CKD). AGE contributes to CKD progression by increasing reactive oxygen species (ROS) generation, up-regulating the expression of the receptor for advanced glycation end products (RAGE), and inducing inflammation and fibrosis, all mechanisms affecting glomerular function. Indeed, this activates a vicious circle that increases AGE and uremic toxin accumulation and oxidative stress and promotes loss of muscle mass and function.

In CKD, AGE accumulation depends on the reduction of their filtration by the kidneys as well as on the increase of their production due to the imbalance between oxidant/antioxidant capacities. The production of AGE is promoted by the uremic milieu, which is characterized by increased oxidative stress and inflammation regardless of the presence of diabetes mellitus (DM) [18,19]. AGE contribute to renal function deterioration [20] and increase cardiovascular risk and mortality in end stage renal disease as well as in kidney-transplanted patients [18,20,21]. The more compromised the renal function, the greater the accumulation of AGE and the amplification of the oxidative stress (Figure 2).

**Figure 3:**
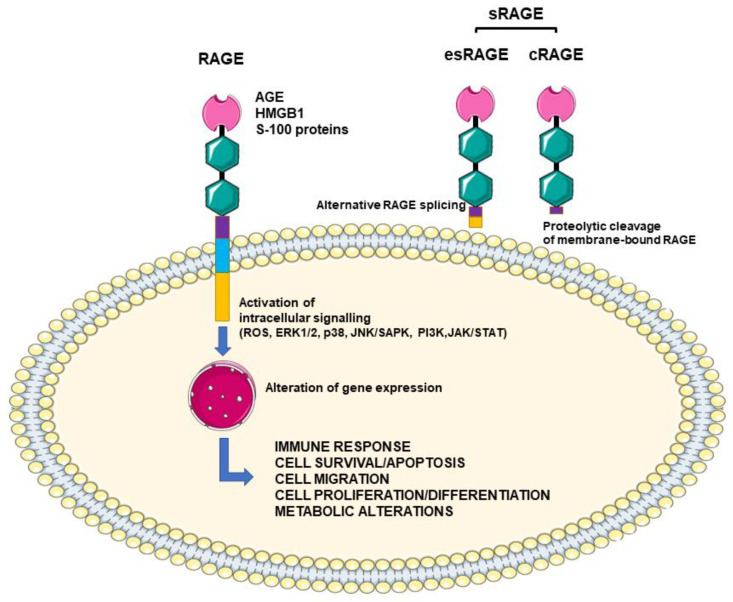
Membrane RAGE (receptor for advanced glycation end products) and its soluble forms esRAGE (endogenous secretory RAGE) and cRAGE (cleaved RAGE). RAGE activation promotes a pro-inflammatory response and additional changes in normal cell functions which induce organ damages. At increasing ligands, membrane RAGE expression is upregulated, and this activates a vicious circle that increases the intracellular synthesis of reactive oxygen species (ROS) and the RAGE-mediated response. Circulating sRAGE, which include esRAGE and cRAGE, can block ligand binding to RAGE, thus playing a role as a decoy receptor. HMGB1, High Mobility Group Box 1; S-100 proteins, a family of 21 proteins structurally similar to calmodulin and considered to be damage-associated molecular pattern molecules; ERK1/2, extracellular signal-regulated protein kinase 1/2; p38, p38 mitogen-activated protein kinase; JNK/SAPK, c-Jun N-terminal kinase/stress-activated protein kinase; PI3K, phosphoinositide 3-kinases; JAK/STAT, Janus kinase/signal transducers and activators of transcription; NF-kB, nuclear factor-kB.

Animal studies supported the detrimental role of AGE in nephropathy: Thickening of the basement membrane and expansion of the mesangial layer have been observed in AGE-injected animals [22]. Increased levels of transforming growth factor β (TGFβ) and reduced nitric oxide production/activity are two key promoters of glomerulosclerosis, which is associated with an increase in oxidative stress [23]. The detrimental effects of AGE may also depend on the activation of the receptor for AGE (RAGE). RAGE is a multiligand receptor of the immunoglobulin superfamily. In addition to AGE, RAGE can also bind other molecules such as HMGB1 (High Mobility Group Box 1) and S-100 proteins, a family of 21 proteins structurally similar to calmodulin and considered as damage-associated molecular pattern molecules. Once activated, RAGE promotes intracellular ROS formation and the activation of multiple intracellular signals such as ERK1/2 (extracellular signal-regulated protein kinase 1/2), p38 (p38 mitogen-activated protein kinase), JNK/SAPK (c-Jun N-terminal kinase/stress-activated protein kinase), PI3K (phosphoinositide 3-kinases) and JAK/STAT (Janus kinase/signal transducers and activators of transcription). The further activation of nuclear factor-kB (NF-kB) induced by these molecules regulates the synthesis of pro-inflammatory cytokines and other mediators and affects cell survival, differentiation and proliferation as well as inducing metabolic changes (Figure 3). Under physiological conditions, RAGE is expressed at low levels, but during conditions of chronic inflammation and oxidative stress, RAGE ligands increase RAGE expression and therefore amplify the inflammatory response [24,25]. This activates a vicious circle that increases the intracellular synthesis of ROS and the RAGE-mediated response (Figure 3).

### 2.2. Defensive Strategies against AGE

sRAGE is the soluble circulating form of RAGE: It blocks ligand binding to AGE, and it can be used as a disease biomarker [26,27,28,29,30]. Indeed, sRAGE is a pool composed by the endogenously secretory form (esRAGE) and the membrane-cleaved form (cRAGE). The first is an alternative splice form of RAGE, and it is considered the real decoy receptor; the latter derives from the proteolytic cleavage of the membrane-bound receptor by metalloproteases, and it is regarded as a surrogate marker of inflammation (Figure 3). RAGE activation has been shown to down-regulate esRAGE and promote metalloproteases to cleavage RAGE into cRAGE. However, in specific pathological conditions characterized by very high AGE levels such as DM and CKD, both forms may increase as potential counter-regulatory mechanisms to protect against AGE and their detrimental effects. Therefore, given that cRAGE and esRAGE are produced by different and independent mechanisms [31,32], their levels can reflect how a disease and/or an intervention can proportionally or disproportionally affect them at the same time.

Besides sRAGE, other defensive strategies exist against AGE. These mechanisms include AGE degradation through endogenous enzymatic glyoxalase-1 and -2 (GLO-1 and GLO-2) and AGE receptor-mediated (AGERs) defense systems. GLO-1 and GLO-2, together with glutathione (GSH), prevent AGE synthesis through the degradation of dicarbonyl compounds [33]. GLO-1 is the rate-limiting enzyme because it catalyzes the first step of detoxification, and its activity is proportional to GSH concentration. Both regulation of gene expression as well as post-translational modifications can affect GLO-1 activity. In particular, hypoxia and inflammation negatively affect GLO1 expression, and the activation of the AGE-RAGE axis also suppresses the expression of GLO-1, thus increasing AGE production and accumulation [34]. AGERs are a receptor family composed of AGER1, AGER2, and AGER3. AGER1, the first discovered, is expressed in most cells. Its role is to accelerate AGE uptake and removal from the circulation, thus blocking AGE binding to RAGE [34]. AGER1 expression increases at increasing AGE levels, but it is downregulated after exposure to persistently elevated levels of AGE. This usually occurs in DM and from ingestion of food containing large amounts of AGE. AGER1 expression has been shown to correlate directly with some intracellular antioxidant systems such as sirtuin-1 (SIRT1), nicotinamide phosphoribosyltransferase (NAMPT), superoxide dismutase 2 (SOD2), and GSH, and negatively with prooxidant pathways such as RAGE, nicotinamide adenine dinucleotide phosphate (NADPH) oxidase and the Src homology/collagen (Shc) adaptor protein p66Shc. AGER1 is therefore important in the maintenance of normal homeostasis [35].

### 2.3. Effect of AGE on Muscle Function

Presently, most of the knowledge on how AGE affect muscle function and about what could be their role in the onset and progression of sarcopenia comes from in vitro works, animal studies, and clinical observations in DM, cancer, and aging [10,11,12]. Healthy muscle contains myoblasts that differentiate into myotubes. Anything promoting myoblast loss and myotube dysfunction can therefore affect skeletal muscle mass and strength, promoting sarcopenia. *C57Bl/6j mice* fed a high-fat, high-sugar diet and *ob/ob mice* fed a standard diet displayed oxidative stress and inflammation and accumulated AGE in muscle fibers and plasma. AGE accumulation can induce myosteatosis, decrease muscle mass, reduce mitochondrial efficiency, and favor the transition of fast-to-low speed muscle fibers [1,36,37]. Increased expression of RAGE on the cellular membrane and activation of the lipogenic pathway SCAP (SREBP cleavage-activating protein)/SREBP (sterol regulatory element binding protein) have been suggested as potential mechanisms linking intracellular AGE accumulation and muscle fiber atrophy [38]. AGE can also directly inhibit myogenic differentiation and promote cellular death, as observed in C2C12 myoblasts [39]. IGF-1 (insulin growth factor-1)/Akt (protein kinase B) signals can attenuate these AGE-related detrimental effects and can therefore represent an interesting therapeutic target to counteract AGE-induced sarcopenia [13,40]. AGE concentration is also associated, both in mouse and human cell lines, with the reduction of myotube diameter and increased expression of MAFbx (muscle atrophy F-box). This last is a protein of the ubiquitin proteasome pathway that can promote intracellular protein degradation in skeletal muscle [39]. Interestingly, the detrimental effects of AGE on myotube atrophy and myogenesis can be blocked by an AGE inhibitor [39]. Chronic activation/overexpression of RAGE was shown to induce muscle wasting and systemic inflammation, while its absence translated into delayed loss of muscle mass and strength [12]. Interestingly, in mice, pharmacologic RAGE inhibition can restore aging-induced alterations of skeletal muscle [41]. These preliminary results suggest that the AGE–RAGE pathway may play a pivotal role in inducing myopathy.

Studies performed in older individuals as well as in DM patients indicated that AGE are inversely associated with muscle strength and mass [11,42]. Loss of appendicular lean mass correlated with levels of pentosidine, an AGE product, which was suggested as a potential biomarker for sarcopenia [10]. In older women, urinary excretion of another AGE, carboxymethyllysine, was negatively associated with grip strength and suggested as a potential tool for sarcopenia screening [43]. Recently, Yabuuchi et al. demonstrated that AGE accumulation in the gastrocnemius muscle of nephrectomized mice associated to morphological abnormalities, capillary rarefaction, and mitochondrial disfunctions [37]. Furthermore, they showed that serum AGE levels were significantly increased according to frailty status and inversely associated with physical performance and physical activity in dialysis patients, and AGE-aptamer treatment improved the deleterious effects of AGE on skeletal muscles. AGE were found to be associated with slowness and weight loss that, along with weakness, exhaustion, and decreased physical activity, are components of frailty. A similar association has been previously observed in older community-dwelling adults, thus confirming that AGE can affect muscle function [44]. Fonseca et al. also observed associations between AGE accumulation and lower muscle stiffness/density in peritoneal dialysis patients [45].

Different mechanisms have been proposed as mediators of AGE detrimental effects. Some of these mechanisms have been briefly indicated in the previous paragraphs. An in-depth description of some of them is reported thereafter instead. Among these potential mechanisms, RAGE activation and inflammation, malnutrition, endothelial disfunction, and connective tissue protein stiffness might really explain how AGE can impair muscle function. However, unlike previous studies which found an association between AGE and weakness in older community-dwelling women [46] and low physical activity in older men [47], Yabuuchi et al. did not find any correlation with these components of frailty [37]. Therefore, this reinforces the need for specific studies on CKD patients. Although the mechanisms leading to sarcopenia and frailty can be the same in different study groups, CKD may have a different background, and the timing and sequence of activation of these detrimental pathways could be different. Given that AGE were found to be inversely correlated with average METs (metabolic equivalent of task) [37], exercise was proposed as a potential strategy to reduce AGE levels and therefore to ameliorate other AGE-related dysfunctions observed in patients with CKD. However, to elucidate this assumption, we need additional studies.

## 3. AGE, Mitochondrial Disfunction, and Sarcopenia

### 3.1. Oxidative Stress and Sarcopenia

**Figure 4:**
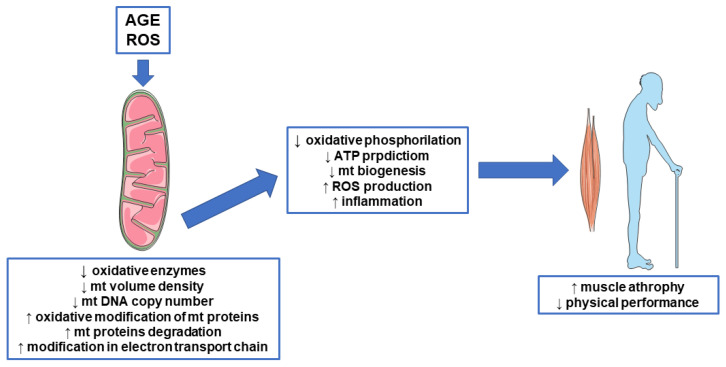
Advanced glycation end products (AGE), mitochondrial disfunction, and sarcopenia. AGE and reactive oxygen species (ROS) can affect mitochondrial function by decreasing oxidative enzymes and inducing oxidative alteration of mitochondrial proteins, which result in protein degradation and loss of function as well as lower mitochondrial (mt) volume density and mitochondrial DNA (mtDNA) copy number, a marker of mitochondrial biogenesis/mass. These mechanisms lead to reduced mitochondrial function, which in turn could induce a state of muscle atrophy.

Mitochondria are involved in many critical cellular processes in skeletal muscle. Indeed, they have a pivotal role in energy supply, ROS production, calcium homeostasis, and regulation of apoptosis [48] (Figure 4). Many studies have previously confirmed the involvement of mitochondria in sarcopenia. Muscle biopsies from CKD patients show decreased oxidative enzymes (cytochrome c oxidase activity and citrate synthase) and mitochondrial proteins [49], lower mitochondrial volume density, and lower mitochondrial DNA (mtDNA) copy number, a marker of mitochondrial biogenesis/mass [50]. Furthermore, at decreasing mtDNA copy numbers in peripheral blood mononuclear cells, the severity of CKD increases. Resting skeletal muscle oxygen consumption and mean mitochondrial coupling ratio were significantly elevated and reduced, respectively, in non-diabetic CKD patients compared to healthy individuals. The finding of a disruption of muscle oxidative phosphorylation in CKD may indicate the activation of processes leading to impaired physical performance. Among different factors, oxidative stress has been suggested to play a major role in disrupting muscle mitochondrial metabolism [51]. Mitochondrial disfunction and alteration in mitochondria biogenesis also increase ROS production and inflammation, which in turn promote the increase of NLRP3 (Nod-like receptor family pyrin domain containing 3) activity that participates in sarcopenia. These bioenergetic alterations were correlated with reduced muscle strength, cardiorespiratory measurements, and muscle function, supporting the involvement of mitochondria in the sarcopenia process [52,53]. Worth noting is the observation that one molecular signature of the transition between healthy to sarcopenic muscle is low mitochondrial bioenergetic capacity [54]. Mitochondria are also the main source of ROS. If from one side, ROS play important roles as signaling molecules, to the other, chronic elevation is pathogenic and causes muscle atrophy [55]. Aging is recognized a driver of ROS accumulation mainly due to the decrease of cellular antioxidant activities that lead to free radicular accumulation [56]. In CKD, we can observe just an imbalance between ROS generation/detoxification. The increased accumulation of AGE due to reduced kidney filtration promotes RAGE activation and inflammation and activates a vicious circle of inflammaging that may further induce mitochondrial dysfunctions.

Although modulation of the AGE/RAGE axis was suggested as an effective strategy to improve mitochondrial damage [57], connections between RAGE, mitochondria and inflammation, and their role in sarcopenia are poorly described but seem very likely in CKD. Therefore, RAGE might play a major role both in age-related sarcopenia as well as in any other condition characterized by an increased activation of this receptor.

### 3.2. AGE and Mitochondrial Proteins

Oxidative stress and products of glycation can affect mitochondrial function and biogenesis also by targeting mitochondrial proteins. These proteins can lose their role and affect mitochondrial functions. After oxidative modification, these proteins can undergo a degradation process involving the activation of specific proteases called mitoproteases. Lon protease homologue 1 (LONP1), the ATP-dependent Clp protease proteolytic subunit (CLPP), the mitochondrial inner membrane protease ATP23, and the intermembrane high-temperature requirement Serine Peptidase 2 (HTRA2/OMI) are members of this group of enzymes [58]. By modulating the activity of mitochondrial proteins by protein processing and degradation, these enzymes regulate mitochondrial stress responses. It has been shown that the reduction of Lon protease due to aging and the invalidation of HTRA2/OMI lead to increased levels of altered mitochondrial proteins and mitochondrial function as well as reduced mitochondrial biogenesis and altered mitochondrial Unfolded Protein Response (mtUPR) activation. This last is a mitochondria-to-nuclear signal transduction pathway which promotes the activation of mitochondrial protective genes to re-establish protein homeostasis within the mitochondrial protein-folding environment [59,60] (Figure 4).

### 3.3. AGE and Mitochondrial Biogenesis

Alteration of mitochondrial biogenesis is another mechanism that can lead to sarcopenia. The nuclear and mitochondrial genomes collaborate with the mitochondrial biogenesis program through the expression of different transcription factors, with peroxisome proliferator-activated receptor-γ coactivator (PGC)-1α being the most important [61]. Lower amount of PGC-1α and mitochondrial proteins in skeletal muscle were reported with aging [62], while PGC-1α overexpression counteracted the negative effects of aging on mitochondrial protein content, thus suggesting that mitochondrial biogenesis can be involved in sarcopenia, as well [63]. Most of the present knowledge comes from studies on aging. Considering that both aging and CKD are characterized by increased circulating levels of AGE, we cannot exclude a direct role of AGE products and AGE-induced oxidative stress and inflammation in deregulating mitochondrial functions in CKD, as well (Figure 4). In fact, it has been shown that oxidative stress and inflammation reduce the expression of PGC-1α and increase the number of muscular mitochondria [64]. Furthermore, indoxyl sulfate, a uremic toxin known to induce oxidative stress, was also found to induce mitochondrial dysfunction via increasing oxidative stress [65].

### 3.4. AGE and Mitochondrial Function

Previous studies demonstrated an existing relationship between AGE and mitochondrial function, although in different settings. Patel et al., by using tendon-derived fibroblasts, demonstrated that AGE negatively affected, in a dose-dependent manner, both cell proliferative capacity and mitochondrial ATP production [66]. Additionally, carboxymethyllysine (CML), an AGE product, induced mitochondrial dysfunction and mitophagy in pancreatic β-cells. The findings from this study suggest that increased concentrations of AGE may damage β-cells and reduce insulin secretion [67]. Mitochondrial abnormalities have been observed to largely contribute to AGE-induced apoptosis in osteoblastic cells, as evidenced by enhanced mitochondrial oxidative stress, conspicuous reduction in mitochondrial membrane potential and ATP production, abnormal mitochondrial morphology, and altered mitochondrial dynamics [68]. ROS produced by mitochondria seem to play a pivotal role in AGE-mediated side effects on mitochondrial function dynamics [9,68]. Patel [66] identified specific targets of the AGE insult among genes involved in the regulation of electron transport complexes and apoptosis. In detail, among genes involved in the regulation of electron transport complexes, a compensatory response to increase mitochondrial complex I has been observed, maybe in an effort to meet energy demands after the AGE insult. Mitochondrial complex III, II, and V (ATP synthase) were reduced. While data genes seem to suggest that AGE may have targeted effects to the electron transport chain, protein analysis revealed that AGE mainly affected only complex III. Furthermore, differently from the gene expression study, complex III protein expression was increased after AGE exposure, not decreased. Timing and dose exposure to AGE could be the main factors affecting these discrepancies among genes and protein studies. Although not conclusive, theses preliminary results suggest how AGE may impact mitochondria function by influencing the electron transport complexes, as well (Figure 4). No data are available on CKD nor its relationship to sarcopenia, but considering that these patients suffer from a long-term exposure to AGE, we could speculate about a potential in vivo detrimental effect of AGE on the respiratory chain.

## 4. AGE, Insulin Resistance, and Sarcopenia

### 4.1. AGE and Inflammation

Abnormalities in insulin signaling can influence the development of sarcopenia. Insulin is not simply involved in carbohydrate metabolism but is an anabolic hormone. Many studies have demonstrated that reduced insulin levels or insulin resistance are associated with protein breakdown, whereas increased insulin levels promote protein synthesis [69]. Siew et al. [70] associated insulin resistance and muscle protein breakdown in hemodialysis (HD) patients. Furthermore, HD patients with DM had a loss of lean body mass greater than those without DM [71]. AGE accumulate in both DM and CKD, and both diseases are characterized by insulin resistance and decreased muscle mass. Could AGE be a link between insulin resistance and muscle loss? Mice fed a high fat, AGE-rich diet showed an impaired insulin sensitivity [72]. In the clinical setting of CKD, it is important to consider that AGE-rich foods can also contribute to increasing AGE accumulation in the blood. The dietary content of AGE determines the serum levels of AGE, inflammatory mediators, and urine AGE levels in both normal subjects and CKD patients. Therefore, consumption of dietary AGE may further promote inflammation, oxidative stress, and insulin resistance in CKD. A systematic review by Rachel E. Clarke et al. [73] compared high AGE intake to low AGE intake in adults with and without obesity, diabetes, or CKD. Studies reported an increase in TNFα, VCAM, and CRP with a high-AGE diet [74]. The effect of the dietary interventions on biomarkers of CKD was not clear. Just in one study was serum creatinine reported with no differences observed [75]. Another study suggested that in well-nourished, predialysis CKD patients, the lowered protein intake adopted by these patients appeared to explain the lower dietary intake of CML, an AGE product, in CKD than in controls [76]. Unfortunately, sarcopenia was not one of the end points explored in these previous studies.

### 4.2. AGE and Genes Involved in Insulin Response

AGE can promote insulin resistance by affecting different cellular mechanisms, including generation of tumor necrosis factor-alpha, direct modification of the insulin molecule thereby leading to its impaired action, generation of oxidative stress, and impairment of mitochondrial function, as examples. Reduced glucose transporter member 4 (GLUT4) translocation and expression are markers of insulin resistance.

**Figure 5:**
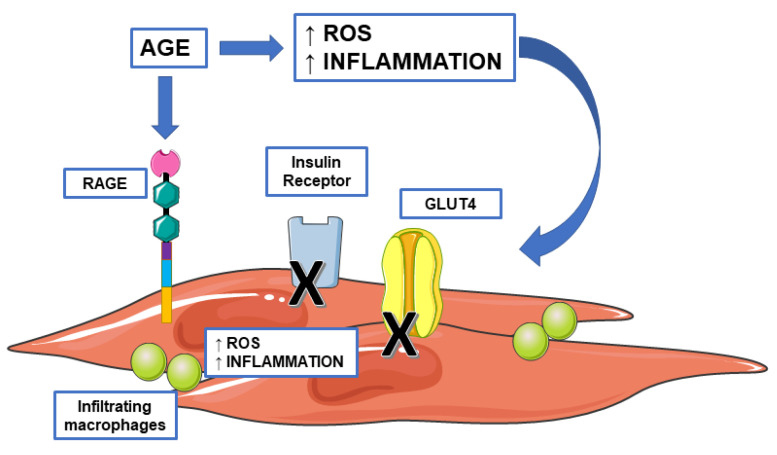
Advanced glycation end products (AGE) and insulin resistance. AGE can affect insulin signaling both directly and indirectly by increasing the circulating levels of reactive oxygen species and pro-inflammatory mediators. AGE can reduce insulin sensitivity by inhibiting the tyrosine kinase activity of the insulin receptor and reducing GLUT4 (Glucose Transporter Type 4) translocation. Accumulation of AGE overstimulates receptors for advanced glycation end product (RAGE) signaling which, along with contributing to fiber damage, recruitment of pro-inflammatory macrophages, and inflammation, may also exacerbate insulin resistance.

The AGE–RAGE axis promotes the generation of ROS and numerous cytokines and chemokines which are known to cause local tissue insulin resistance [77] through mechanisms including inhibition of tyrosine kinase activity of the insulin receptor and down regulation of GLUT4 and other genes involved in insulin responsiveness [78]. Macrophages are inflammatory cells known to express RAGE. Skeletal muscle accommodates both resident macrophages along with macrophages that can infiltrate the tissue in the presence of specific chemotactic signals. Studies performed in specific muscle diseases such as muscular dystrophy suggested that the accumulation of RAGE ligands overstimulated RAGE signaling, which resulted in macrophage infiltration, secretion of pro-inflammatory cytokines, and oxidative stress. Along with contributing to fiber damage [79], recruitment of pro-inflammatory cells, and inflammation, RAGE may also exacerbate insulin resistance [80]. Treatment of L6 skeletal muscle cells with glycated albumin affected the signaling of insulin-induced insulin receptor substrate (IRS) 1 and 2 through a PKCα (protein kinase C alpha)-mediated mechanism [81]. AGE might also affect insulin sensitivity through RAGE activation and ROS overproduction [82]. Although this mechanism was primarily described in adipocytes, we cannot exclude the activation of the same pathway in muscle cells, as well. AGE have been shown to increase oxidative stress and to activate endoplasmatic reticulum and inflammatory stress in skeletal muscle. These events culminate with the repression of GLUT4 expression [83] (Figure 5). The strong link between AGE levels and insulin sensitivity comes from a study by Hoffman, which demonstrated that db/db mice fed a high-AGE diet had increased plasma AGE levels and insulin resistance compared to mice fed a low-AGE diet [84].

Presently, data linking AGE, insulin-resistance, and sarcopenia in CKD patients not yet on dialysis are lacking. However, the studies performed up to now in other clinical conditions characterized by high levels of AGE confirmed that, along with exerting a direct damaging effect on muscle fibers, AGE could induce loss of muscle mass by promoting insulin resistance. Blocking AGE might reduce these effects, but this needs to be explored in CKD.

## 5. Therapeutic Intervention: Srage, Anti-Advanced Glycation End Products Agents, and Therapies Targeting Mitochondria

sRAGE can play a protective role against AGE and other RAGE ligands by working as a scavenger receptor. The association between sRAGE and sarcopenia in human has been poorly explored. Kim et al. showed the independent association of sRAGE with the presence of low muscle mass in a Korean population [85]. These data suggest a potential protective role of sRAGE against sarcopenia. However, no data exist about sRAGE and sarcopenia in CKD. Considering that sRAGE accumulate in CKD along with AGE at decreasing of kidney function, we should look at sRAGE in CKD more as a potential biomarker of sarcopenia than as a possible therapeutic strategy. Therefore, also this topic deserves further investigations.

Considering the detrimental effects of AGE, any strategy that can decrease their levels might have beneficial effects in different clinical settings. Current therapeutic options to reduce AGE include AGE cross-link breakers, AGE inhibitors, RAGE antagonists, nutrition, and phytotherapy. However, just a few of these have been clinically evaluated and along with potential beneficial effects, side effects have been described for some [86]. Unfortunately, until now, no specific effects of these treatments on sarcopenia have been investigated. The results obtained until now by in vitro and in vivo studies confirmed that RAGE inhibitors are a useful tool in the management of inflammation. In fact, RAGE inhibition reduces NF-κB activation, cytokine production, and the over-expression of RAGE. Considering the involvement of RAGE in many diseases, these drugs could find applications in different fields including CKD-related sarcopenia [87].

Resistance exercise training has been shown to reverse sarcopenia and improve mitochondrial function in aging muscle. Six months of resistance exercise training reversed both at the phenotypic and transcriptome level mitochondrial impairment and muscle weakness [88]. This kind of training was also effective in improving muscle strength and mitochondrial function as well as reducing oxidative stress [89]. In CKD patients, resistance training counteracted the catabolism of a low-protein diet, increased muscle accretion, and reversed muscle weakness [90,91]. Furthermore, mtDNA copy number significantly increased after 12 weeks of high-intensity resistance exercise training compared to controls and correlated with skeletal muscle mass measured using cross-sectional areas of type I and II fibers [92].

Another approach is focused on reducing oxidative stress. As previously discussed, oxidative stress may be a causative factor that can promote sarcopenia, both directly and indirectly, by increasing AGE formation. It is also clear that oxidative stress is the result of mitochondrial disfunction and activation of the AGE/RAGE axis. Therefore, it is not surprising that antioxidants such as l-carnitine, Coenzyme Q10, and vitamin E, are the main molecules utilized to reduced ROS production by dysfunctional mitochondria. Other pharmacological drugs with antioxidant side effects include N-acetylcysteine, carvedilol, and captopril as well as mitochondria-targeted molecules that achieve higher concentration within mitochondria such as MitoE and MitoQ. Unfortunately, as previously discussed for some anti-AGE products, most of these compounds have not been evaluated for their potential protective effect on muscle function, particularly in CKD [93,94].

## 6. Conclusions

AGE play a detrimental role in sarcopenia, as demonstrated in many conditions characterized by increased AGE levels. AGE can affect muscle health through different mechanisms that work simultaneously and speed up the process of muscle deterioration. Up until now, there have been just a few preliminary results suggesting that AGE may induce sarcopenia in CKD. New studies are therefore needed to investigate the mechanisms linking AGE to sarcopenia and to identify new potential markers of disease progression and/or therapeutic targets to prevent the onset and/or the progression of muscle wasting in CKD.

## Data Availability

Not applicable.
